# CT-guided spinal nerve root adhesiolysis for refractory atypical symptoms in cervical disc herniation: a retrospective cohort study

**DOI:** 10.3389/fmed.2026.1755003

**Published:** 2026-05-22

**Authors:** Qiong Xian, Yang Mao-jiang, Yang Hong-ying, Yang Han-feng, Xu Xiao-xue, Du Yong

**Affiliations:** 1 Medical Education Center of Jinan University, Guangzhou, China; 2 Department of Pain, Affiliated Hospital of North Sichuan Medical College, Nanchong, Sichuan, China; 3 Department of Radiology, Affiliated Hospital of North Sichuan Medical College, Nanchong, Sichuan, China

**Keywords:** atypical symptoms, cervical disc herniation, CT-guidance, dizziness, ozone, spinal nerve adhesiolysis, tinnitus

## Abstract

**Objective:**

To evaluate the clinical efficacy and safety of CT-guided spinal nerve root adhesiolysis in treating refractory atypical symptoms—specifically dizziness and tinnitus—in patients with cervical disc herniation (CDH).

**Methods:**

This retrospective cohort study included 256 patients with CDH presenting with persistent atypical symptoms who had failed conservative therapy for at least 3 months. All patients underwent CT-guided periradicular adhesiolysis involving the injection of an ozone-oxygen mixture, normal saline, and corticosteroids. Primary outcomes included symptom alleviation rates and functional improvements assessed via the Dizziness Handicap Inventory (DHI), Tinnitus Handicap Inventory (THI), Visual Analog Scale (VAS) for pain, and Neck Disability Index (NDI) at 1, 3, 6, and 12 months post-procedure.

**Results:**

At the 12-month follow-up, the intervention demonstrated significant efficacy. The alleviation rate for dizziness (baseline prevalence 85.2%) was 80.3%, and for tinnitus (baseline prevalence 68.8%) was 75.6%. Statistically significant improvements were observed in all functional scores compared to baseline (*P* < 0.001). Specifically, DHI scores decreased from 65.2 ± 12.5 to 18.7 ± 6.0, and THI scores decreased from 58.7 ± 11.3 to 18.0 ± 5.8. 8 Subgroup analysis of patients with isolated dizziness or tinnitus confirmed efficacy independent of neck pain relief. The procedure showed a favorable safety profile with no serious adverse events.

**Conclusion:**

For patients with CDH-related atypical symptoms refractory to conservative management, CT-guided spinal nerve root adhesiolysis is a safe and effective minimally invasive intervention. It provides sustained functional improvement and represents a vital therapeutic option for this challenging patient population.

## 1 Introduction

Cervical disc herniation (CDH) is a prevalent degenerative spinal disorder, typically characterized by radicular symptoms including neck pain, cervicobrachialgia, and sensorimotor deficits (1). However, beyond these typical presentations, a significant cohort of patients develops a spectrum of non-radicular “atypical symptoms” (2–4), such as dizziness, tinnitus, visual disturbances, nausea, and palpitations. These manifestations are frequently overlooked or misattributed to other systemic disorders, significantly impairing patients’ quality of life.

The pathogenesis of these atypical symptoms is multifactorial, primarily attributed to two key mechanisms: vertebrobasilar insufficiency and cervical sympathetic nervous system dysfunction (5, 6). Mechanical irritation or compression of the vertebral artery by a herniated disc or osteophytes can lead to cerebral hypoperfusion, manifesting as dizziness, vertigo, and visual disturbances. Concurrently, inflammation-mediated irritation of regional sympathetic nerve fibers can provoke autonomic dysregulation, resulting in symptoms such as palpitations, gastrointestinal distress, and tinnitus (4).

Despite the clinical burden of these symptoms, a therapeutic gap remains. Conventional conservative treatments often provide insufficient relief, while open surgeries, such as anterior cervical discectomy and fusion (ACDF), carry substantial risks that may not be justifiable for patients without severe myelopathy. Current minimally invasive options, such as epidural steroid injections, primarily address inflammation but may fail to resolve mechanical adhesions.

CT-guided spinal nerve root adhesiolysis is a precise technique designed to address this gap. It delivers a multi-modal therapeutic mixture—comprising ozone, normal saline, and an anti-inflammatory solution—directly to the pathologic site. This approach combines the anti-inflammatory and dehydrating effects of ozone with the mechanical lavage of adhesions (7–9). We hypothesized that this intervention would provide significant and durable relief for patients with CDH-related atypical symptoms refractory to conservative management. This study retrospectively evaluates the clinical efficacy and safety of this technique in a large cohort of 256 patients.

## 2 Materials and methods

### 2.1 Study design and ethics

This retrospective cohort study was approved by the Institutional Ethics Committee (Approval No.: 2025ER355-1). Informed consent was waived due to the retrospective nature of the analysis and the use of de-identified data.

### 2.2 Patient selection

We reviewed clinical records of 1,054 patients treated for cervical spine diseases between January 1, 2020, and December 31, 2023. A total of 256 patients met the inclusion criteria.

#### 2.2.1 Inclusion criteria

(a) Diagnosis of CDH confirmed by MRI or CT. (b) Presence of at least one atypical symptom (dizziness, tinnitus, visual disturbances, nausea, palpitations, or gastrointestinal symptoms). (c) Persistent symptoms refractory to standardized conservative treatments for at least 3 months. Conservative treatments considered included: (i) oral medications (nonsteroidal anti-inflammatory drugs, muscle relaxants, and neuropathic pain agents such as gabapentin); (ii) physical therapy (cervical traction, strengthening exercises, postural training); (iii) short-term cervical collar immobilization (typically 1–2 weeks); and (iv) activity modification with ergonomic advice. The duration of conservative treatment ranged from 3 to 12 months prior to referral, and all patients had failed to achieve adequate symptom relief despite documented compliance. d) Clinical manifestations consistent with radiologically confirmed nerve root compression or irritation.

#### 2.2.2 Exclusion criteria

To ensure diagnostic accuracy, we rigorously excluded atypical symptoms attributable to other medical conditions. All patients underwent multidisciplinary specialist evaluations prior to enrollment to rule out non-cervical etiologies. Specifically, patients with dizziness/vertigo were assessed by otolaryngology (including audiometry and vestibular function tests when indicated); those with palpitations or unexplained dizziness underwent cardiology evaluation (ECG, Holter monitoring); patients with nausea or epigastric discomfort received gastroenterology consultation (with endoscopy if indicated); and those with visual disturbances were examined by ophthalmology. Only after these evaluations confirmed that symptoms were likely cervicogenic—with no identifiable primary non-cervical cause—were patients included in the study. Patients with a history of cervical surgery, severe neurological deficits, or coagulopathy were also excluded. Rigorous exclusion of these alternative etiologies was a critical step in this study to ensure that observed treatment effects could be accurately attributed to the intervention for CDH.

### 2.3 Interventional procedure

#### 2.3.1 Preoperative preparation

A comprehensive preoperative assessment was conducted for each patient. Detailed explanations regarding the procedure were provided to the patients and their families, ensuring informed consent was obtained. Patients were required to fast for at least 6 h before the procedure.

#### 2.3.2 Medication management

To eliminate potential confounding by systemic anti-inflammatory medications, a standardized protocol was followed. All patients were required to discontinue oral nonsteroidal anti-inflammatory drugs (NSAIDs) and corticosteroids at least 1 week prior to the procedure. Post-procedure, no routine oral NSAIDs or corticosteroids were prescribed. Acetaminophen (paracetamol) was permitted as a rescue analgesic for mild discomfort if needed, but its use was minimal and documented. This uniform protocol ensured that the observed therapeutic effects could be attributed primarily to the CT-guided adhesiolysis intervention rather than concurrent pharmacotherapy.

#### 2.3.3 Intraoperative procedure: CT-guided spinal nerve root adhesiolysis

**FIGURE 1:**
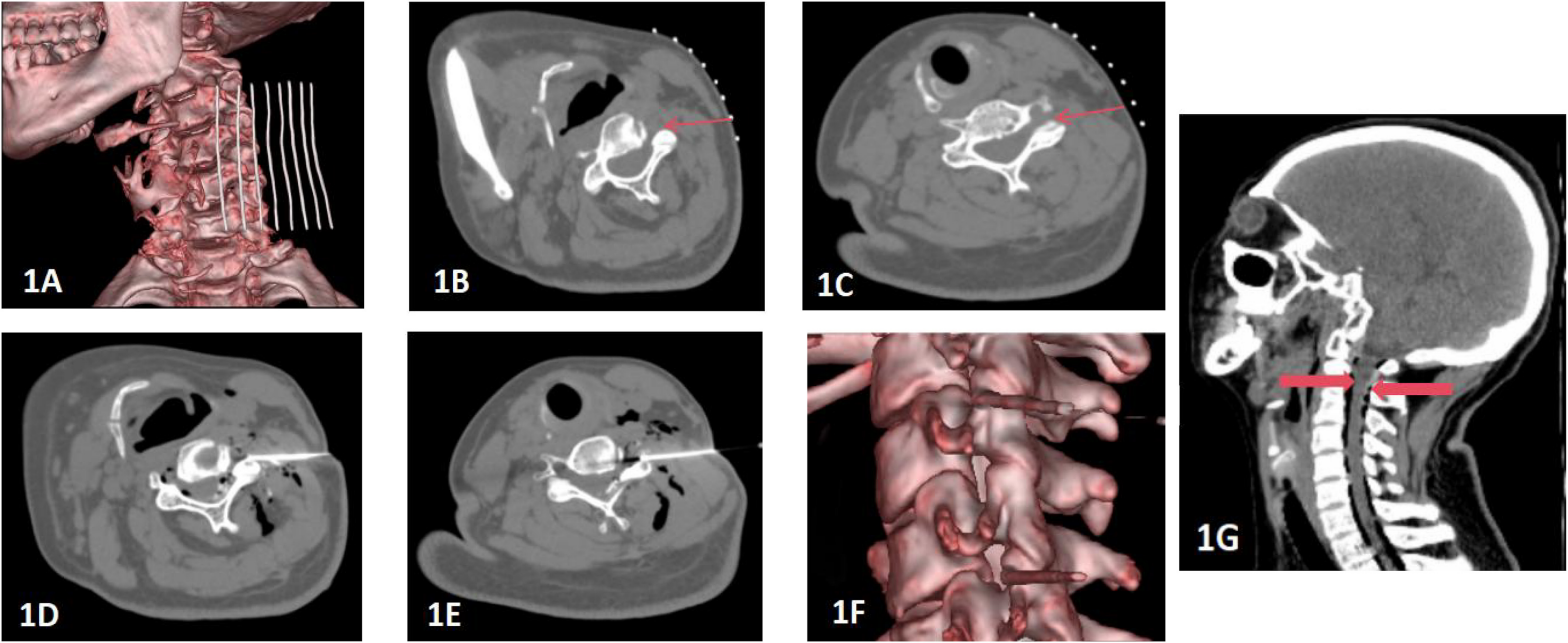
**(A–C)** Preoperative positioning: The patient was positioned supine on the operating table, with the head turned toward the contralateral side. A metal grid marker was placed on the neck to facilitate precise targeting, focusing on the articular pillar surfaces at the targeted disc levels. **(D–F)** Needle Placement and Injection: The needle was precisely inserted along a pre-established pathway targeting the surface of the superior articular process. A therapeutic mixture consisting of 10 ml of ozone blended with physiological saline, complemented by 1 ml of contrast agent, was meticulously injected at each designated site. Multiplanar 3D CT reconstruction techniques were utilized to confirm the accurate positioning of the needle tip at the level of the intervertebral foramen. **(G)** Postoperative CT Scan Reconstruction: The sagittal view of the cervical spine after surgery exhibits a satisfactory dispersion of ozone throughout the spinal canal. Gas shadows, clearly surrounding the dura mater, are marked by thick red arrows. There are no indications of hemorrhage detected in the visualized areas.

All procedures were performed under CT guidance. Patients were positioned supine with the neck exposed. A metal grid marker was used to facilitate precise targeting of the articular pillar surfaces at the affected levels. Under local anesthesia (1% lidocaine), a 22G needle with a coaxial cannula was advanced via an anterolateral approach into the intervertebral foramen. Upon confirmation of needle tip placement by CT reconstruction, a therapeutic mixture was injected sequentially: (1) Ozone–oxygen mixture: 10 mL of medical-grade ozone–oxygen gas (ozone concentration: 30 μg/mL) was injected for anti-inflammatory and dehydrating effects. This concentration refers to the ozone content within the gas mixture prior to injection. (2) Normal saline: 10 mL was injected separately for mechanical adhesiolysis and lavage of inflammatory mediators. (3) Anti-inflammatory Solution: A mixture of betamethasone (5 mg) and lidocaine for immediate analgesia and inflammation suppression (Figure 1).

#### 2.3.4 Postoperative management

Patients were monitored for vital signs and neurological status and were typically discharged on the following day with instructions for neck functional exercises and scheduled follow-up visits.

### 2.4 Outcome measures

Primary outcome: Alleviation rate of atypical symptoms at 1, 3, 6, and 12 months. Alleviation was defined as a ≥ 50% reduction in symptom severity or complete resolution.

Secondary outcome: Functional scores including the Dizziness Handicap Inventory (DHI) (10, 11), Tinnitus Handicap Inventory (THI) (12), Visual Analog Scale (VAS) for neck pain (13), and Neck Disability Index (NDI) (14).

Safety: Incidence of intraoperative and postoperative complications.

### 2.5 Statistical analysis

All data were analyzed using SPSS (version 26.0). Continuous variables are presented as mean ± standard deviation (Mean ± SD), and categorical variables as frequency (n) and percentage (%). Paired *t*-tests or Wilcoxon signed-rank tests were used for pre- and post-procedure comparisons of continuous variables. Chi-square or Fisher’s exact tests were used for categorical variables. A *P*-value < 0.05 was considered statistically significant.

## 3 Results

### 3.1 Baseline characteristics

**FIGURE 2:**
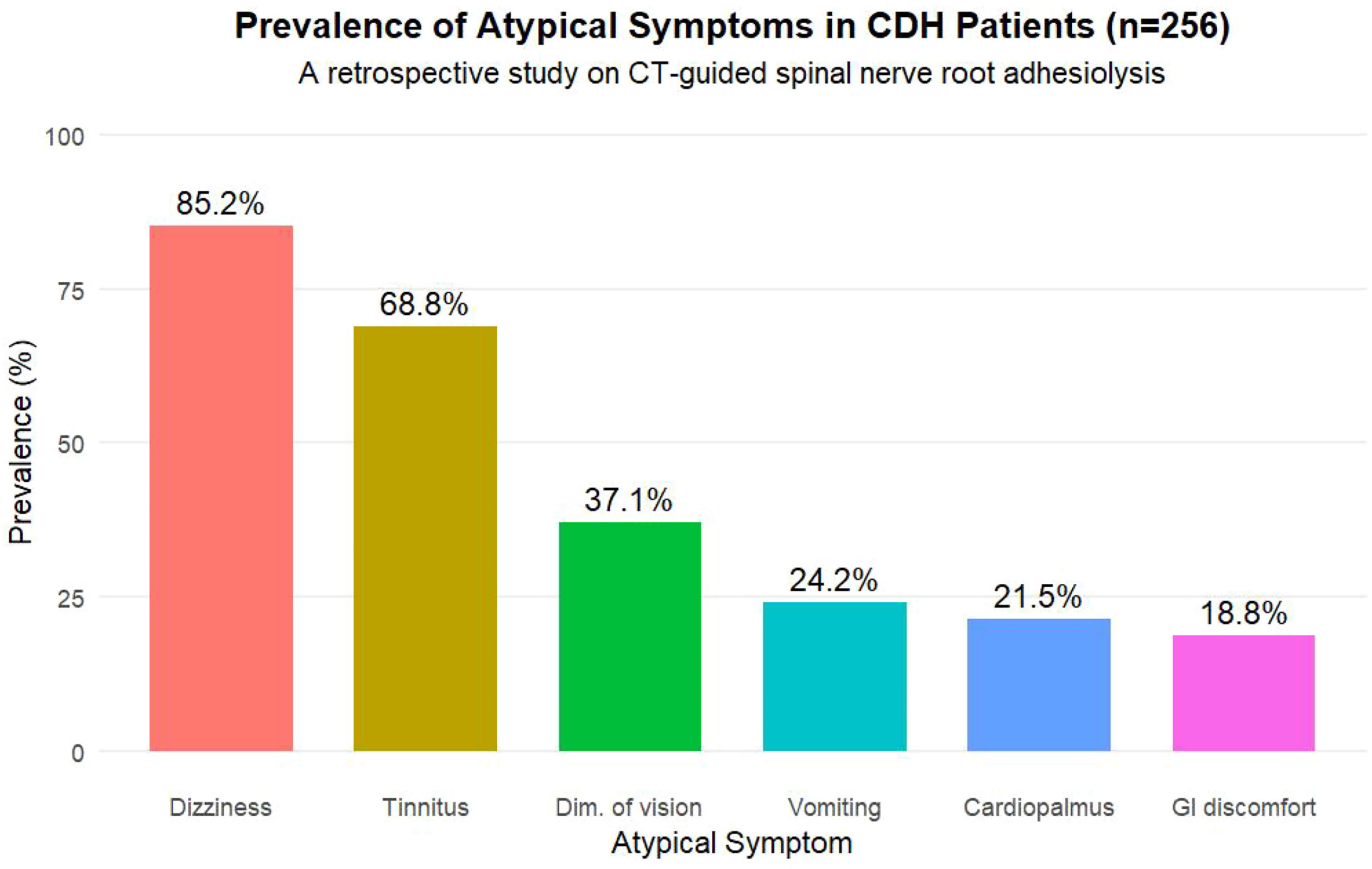
Baseline distribution of atypical symptoms in the study cohort (*n* = 256). Illustrates the prevalence (%) of refractory atypical symptoms at enrollment. Percentages sum to > 100% due to frequent symptom co-occurrence.

The cohort consisted of 256 patients (135 males, 52.7%) with a mean age of 52.3 ± 9.8 years and a mean symptom duration of 18.5 ± 7.2 months. Dizziness (85.2%) and tinnitus (68.8%) were the most prevalent atypical symptoms. Baseline functional scores indicated severe impairment (DHI: 65.2 ± 12.5; THI: 58.7 ± 11.3). Detailed characteristics are in Figure 2 and Table 1.

**TABLE 1:** Baseline characteristics of patients concomitant atypical symptoms.

Characteristic	Value
Numbers (n)	256
Age (years)	52.3 ± 9.8
Gender (n, %)	Male: 135 (52.7%)
	Female: 121 (47.3%)
Duration (months)	18.5 ± 7.2
Symptom (n, %)	
Dizziness	218 (85.2%)
Tinnitus	176 (68.8%)
Visual disturbances OR blurred vision	95 (37.1%)
Vomiting	62 (24.2%)
Palpitations	55 (21.5%)
Gastrointestinal symptoms	48 (18.8%)
Baseline scale scores	
DHI	65.2 ± 12.5
THI	58.7 ± 11.3
VAS (Neck and shoulder pain)	6.8 ± 1.5
NDI	48.9 ± 9.2

Symptom overlap: Percentages total > 100% due to frequent co-occurrence of multiple atypical symptoms in individual patients (e.g., a patient may simultaneously report dizziness, tinnitus, and palpitations). Scale score interpretation: Baseline scores indicate severe functional impairment (DHI > 60: severe dizziness handicap; THI > 56: catastrophic tinnitus impact; NDI > 40: severe neck disability).

### 3.2 Symptom alleviation rates

**FIGURE 3:**
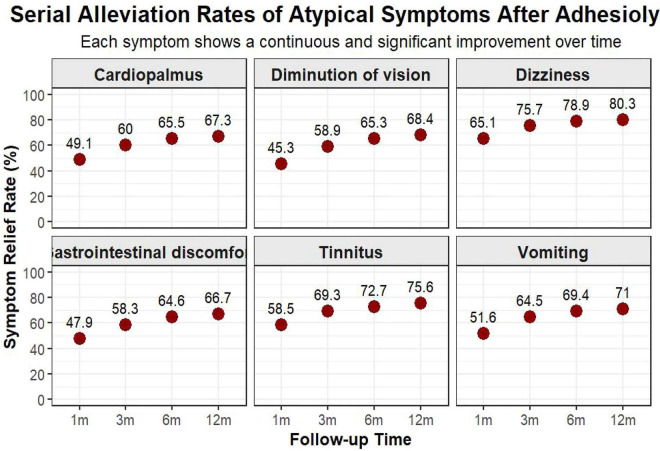
Temporal improvement in atypical symptoms following CT-guided adhesiolysis. Depicts the alleviation rates (%) of refractory atypical symptoms over the 12-month follow-up period.

The intervention demonstrated sustained efficacy over the 12-month follow-up. The alleviation rate for dizziness improved from 65.1% at 1 month to 80.3% at 12 months. Similarly, tinnitus relief rates increased from 58.5 to 75.6% (Table 2). Other symptoms, including visual disturbances and palpitations, also showed progressive improvement rates of 68.4 and 67.3%, respectively, at 1 year (Figure 3).

**TABLE 2:** Serial alleviation rates of atypical symptoms following CT-guided adhesiolysis.

Atypical symptoms	Numbers (n)	Post-1 months relief-rate (%)	Post-3 months	Post-6 months	Post-12 months
Dizziness	218	65.1	75.7	78.9	80.3
Tinnitus	176	58.5	69.3	72.7	75.6
Visual disturbances OR Blurred vision	95	45.3	58.9	65.3	68.4
Vomiting	62	51.6	64.5	69.4	71
Palpitations	55	49.1	60	65.5	67.3
Gastrointestinal symptoms	48	47.9	58.3	64.6	66.7

Symptom remission rate was defined as a reduction of ≥ 50% in symptom severity from baseline or complete resolution post-surgery; Percentages exceed 100% due to overlapping symptoms (e.g., dizziness, tinnitus, nausea) in some patients.

### 3.3 Functional improvements

**FIGURE 4:**
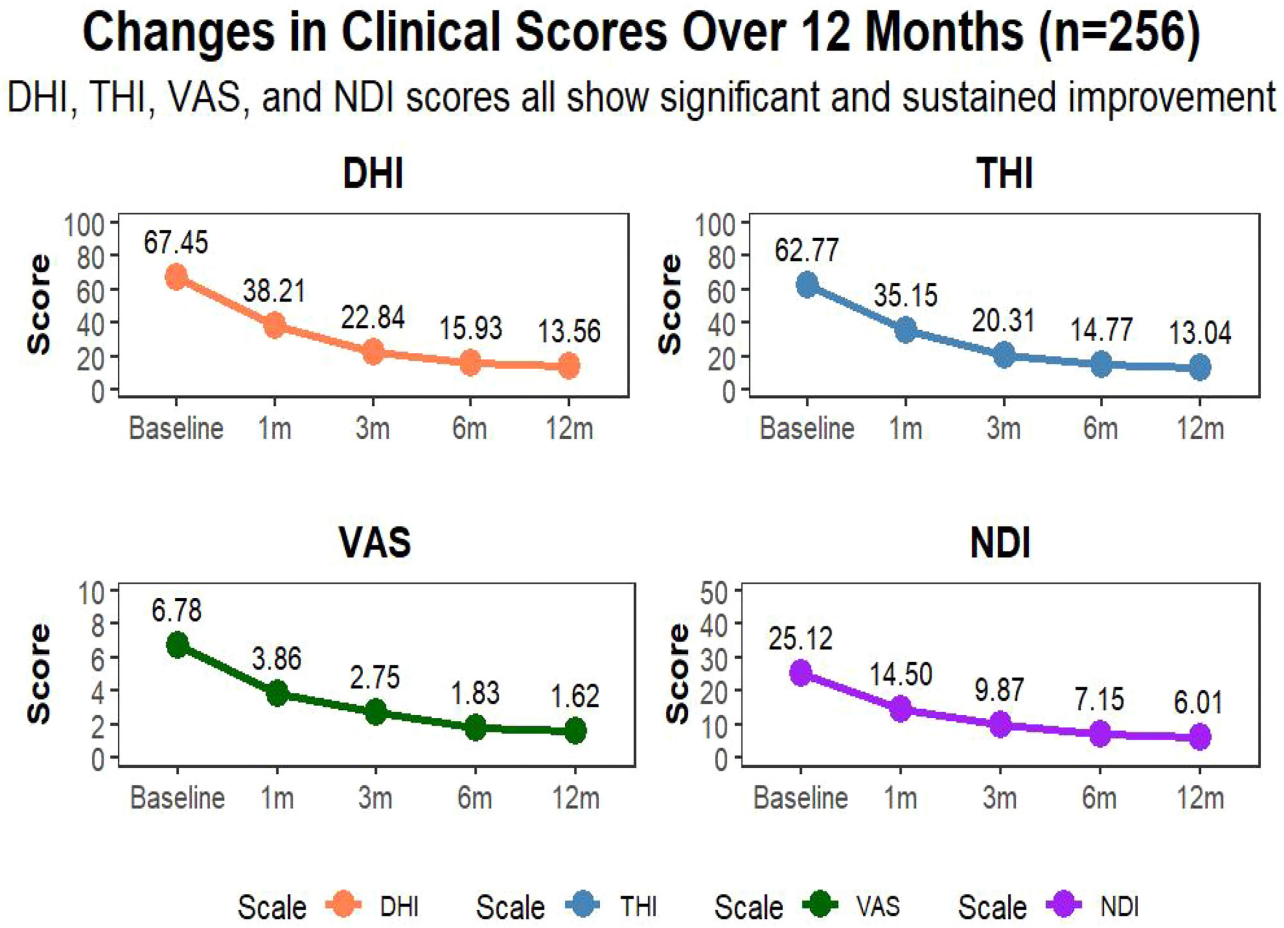
Longitudinal improvement in functional outcome scores after CT-guided adhesiolysis. Demonstrates the significant reduction in DHI, THI, VAS, and NDI scores across the 12-month follow-up period (all *P* < 0.001 vs. baseline), indicating sustained functional recovery.

Significant reductions in all outcome scores were observed at all follow-up intervals (*P* < 0.001). By month 12, the mean DHI score dropped to 18.7 ± 6.0, and the THI score to 18.0 ± 5.8. The VAS score for pain decreased from 6.8 ± 1.5 to 1.3 ± 0.5, and NDI scores improved from 48.9 ± 9.2 to 14.0 ± 4.8, reflecting substantial functional recovery (Figure 4 and Table 3).

**TABLE 3:** Scale scores before and after CT-guided spinal nerve root adhesiolysis.

Scale	Baseline	Post-1 month	Post-3 months	Post-6 months	Post-12 months	*P*-value
DHI	65.2 ± 12.5	38.5 ± 9.1	25.3 ± 7.8	20.1 ± 6.5	18.7 ± 6.0	< 0.001
THI	58.7 ± 11.3	35.1 ± 8.7	23.9 ± 7.1	19.5 ± 6.2	18.0 ± 5.8	< 0.001
VAS	6.8 ± 1.5	3.2 ± 1.0	2.1 ± 0.8	1.5 ± 0.6	1.3 ± 0.5	< 0.001
NDI	48.9 ± 9.2	28.7 ± 7.5	19.5 ± 6.3	15.2 ± 5.0	14.0 ± 4.8	< 0.001

All scale scores (DHI, THI, VAS, NDI) showed statistically significant improvements at all postoperative time points compared to baseline (*P* < 0.001). The reductions in scores reflect substantial functional and symptom improvement, consistent with the clinically observed “excellent effect.”

### 3.4 Subgroup analysis: isolated symptoms

**FIGURE 5:**
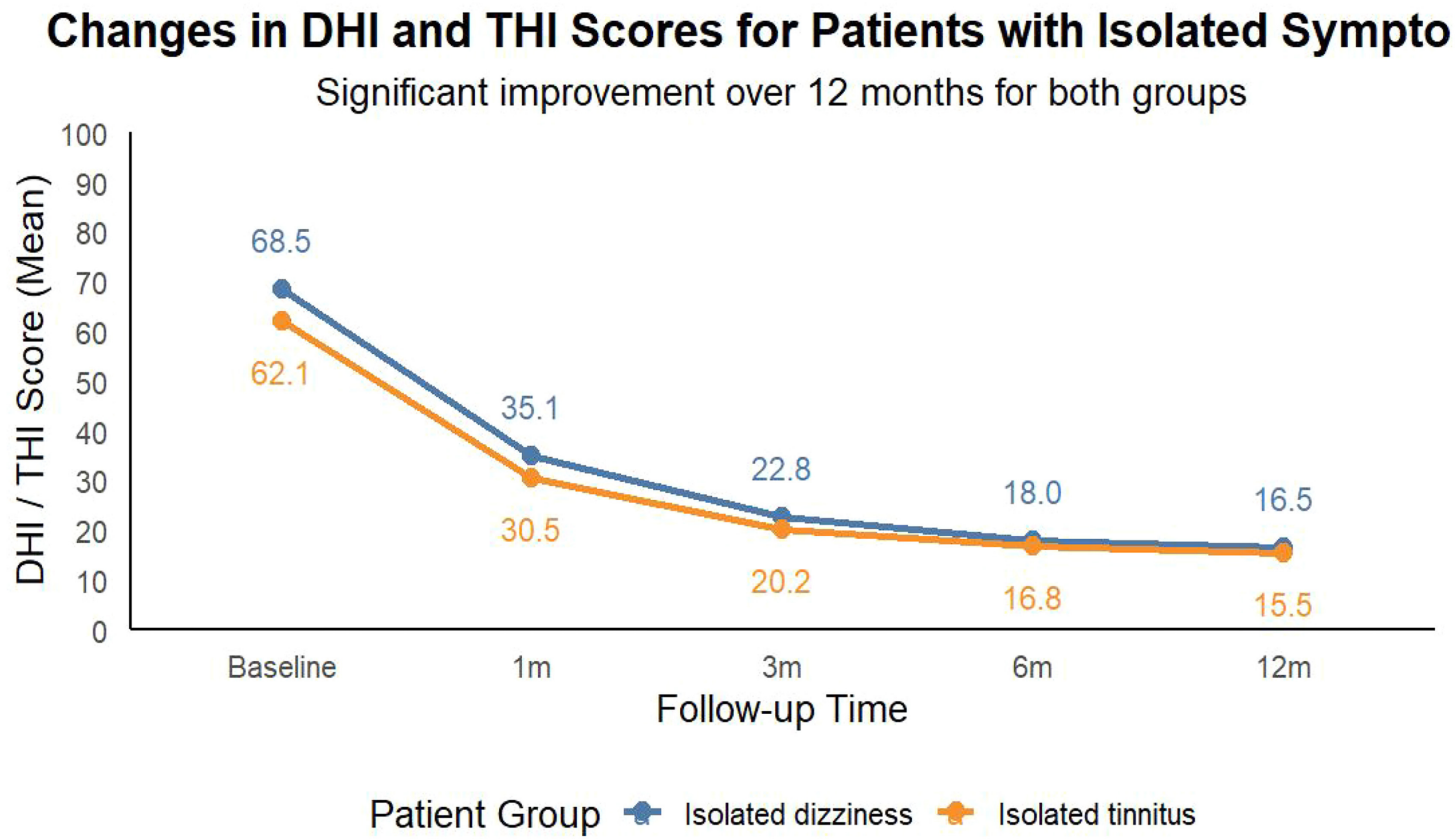
Efficacy of CT-guided adhesiolysis in isolated atypical symptoms. Demonstrates significant improvement in DHI (isolated dizziness) and THI (isolated tinnitus) scores over 12 months in patients without concomitant neck/shoulder pain (all *P* < 0.001 vs. baseline).

In patients presenting with isolated dizziness (*n* = 18) or isolated tinnitus (*n* = 12) without concomitant neck pain, statistically significant improvements in DHI and THI scores were achieved (*P* < 0.001). This confirms the procedure’s efficacy for cervicogenic symptoms independent of somatic pain relief (Figure 5 and Table 4).

**TABLE 4:** Efficacy in patients with isolated dizziness and isolated tinnitus.

Patient group	Numbers(n)	Baseline DHI/THI	Post-1 m	Post-3 m	Post-6 m	Post-12 m	*P*-value
Isolated dizziness	18	68.5 ± 10.2	35.1 ± 8.5	22.8 ± 6.9	18.0 ± 5.5	16.5 ± 5.0	< 0.001
Isolated tinnitus	12	62.1 ± 9.5	30.5 ± 7.8	20.2 ± 6.0	16.8 ± 5.2	15.5 ± 4.8	< 0.001

Data represent patients with isolated dizziness (*n* = 18) or isolated tinnitus (*n* = 12), without concomitant neck/shoulder pain or upper limb symptoms. DHI/THI scores showed statistically significant improvements at all postoperative time points (*P* < 0.001), confirming efficacy in isolated atypical symptoms.

### 3.5 Safety profile

The overall complication rate was 14.6%, consisting entirely of mild and transient events. Common issues included injection site pain (5.9%) and transient neck discomfort (4.7%). No serious adverse events, such as nerve injury, infection, or stroke, were observed (Table 5).

**TABLE 5:** Postoperative complication rates and management (*n* = 256).

Complications/adverse events	Number of cases(n)	Incidence rate (%)
Pain at puncture site	15	5.9
Postoperative transient neck discomfort	12	4.7
Transient dizziness worsens	5	2
Local hematoma	3	1.2
Neuropathic irritation symptoms	2	0.8
Infection	0	0
Severe nerve damage	0	0

All complications were mild and transient, with no severe adverse events (e.g., nerve injury, infection, hemorrhage) observed. Postoperative transient symptoms (e.g., neck discomfort, dizziness exacerbation) resolved spontaneously within days. No stroke or permanent neurological deficits were recorded during the 12-month follow-up.

## 4 Discussion

### 4.1 Overview of key findings

This retrospective study evaluated the clinical efficacy and safety of CT-guided spinal nerve root adhesiolysis in 256 patients with cervical disc herniation (CDH) presenting with refractory atypical symptoms. The results demonstrate that this minimally invasive interventional technique significantly alleviates a range of atypical symptoms, including dizziness, tinnitus, visual disturbances, nausea, palpitations, and gastrointestinal symptoms. Concurrently, it substantially reduced neck pain and functional disability. Statistically significant improvements in all outcome scale scores (DHI, THI, VAS, NDI) were sustained throughout the 12-month follow-up period. Furthermore, the procedure exhibited a favorable safety profile with a low incidence of predominantly minor and transient complications. These findings provide valuable clinical evidence supporting the use of this technique for managing atypical symptoms in CDH patients.

### 4.2 Interpretation of findings and comparison with existing literature

Cervical disc herniation (CDH) is a prevalent degenerative spinal disorder. While typical symptoms such as neck pain and radiculopathy are well-recognized (1), CDH patients frequently experience a spectrum of non-specific or atypical symptoms including dizziness, tinnitus, visual disturbances, nausea, palpitations, and gastrointestinal symptoms. These symptoms are often overlooked or misdiagnosed, significantly impairing quality of life (2–4). This study uniquely focuses on this specific and challenging patient population, employing a precise minimally invasive interventional technique. With a substantial cohort size of 256 patients exhibiting these specific manifestations, our findings provide robust evidence addressing a significant gap in the current literature.

Current research on interventions for CDH with concomitant atypical symptoms remains limited, with most studies focusing on evaluating the efficacy of surgical approaches. Various surgical techniques are employed based on patient factors and lesion characteristics. Anterior cervical discectomy and fusion (ACDF) has been shown to achieve good to excellent overall improvement in sympathetic symptoms in approximately 80.6% of patients at mid-term follow-up (15). However, its efficacy varies across symptom types: while dizziness, headache, nausea, and palpitations consistently improve (16, 17), tinnitus and blurred vision often do not reach statistical significance (16, 17). Total disc replacement (TDR) preserves segmental motion and has demonstrated mean improvement rates of 71.2% for vertigo, 62.3% for headache, and 80.3% for nausea/vomiting based on numerical rating scale scores (18); notably, tinnitus improved by only 39.8%, and gastrointestinal discomfort showed no significant change (18). Posterior decompression via double-door laminoplasty, indicated for multilevel disease, also alleviates most atypical symptoms, with reported score reductions of 66.0% for vertigo, 64.9% for headache, and 75.7% for nausea, but again tinnitus (27.5%) and hypomnesia showed limited improvement (19).

In this context, our CT-guided spinal nerve root adhesiolysis technique achieved alleviation rates (defined as ≥ 50% symptom reduction or complete resolution) of 80.3% for dizziness, 75.6% for tinnitus, and 68.4% for visual disturbances at 12 months. These results compare favorably with surgical outcomes, particularly for tinnitus, where our technique demonstrated superior efficacy relative to both ACDF [non-significant improvement in most series (16, 17)] and motion-preserving procedures [TDR: 39.8% (18); laminoplasty: 27.5% (19)]. This advantage may stem from the multimodal mechanism of adhesiolysis, which directly addresses perineural inflammation and sympathetic irritation at the affected nerve root level—a more targeted approach than indirect decompression achieved by surgery. Moreover, as a minimally invasive procedure with a favorable safety profile, our technique offers a valuable therapeutic option for patients with refractory atypical symptoms who may not be candidates for or wish to avoid major surgery.

### 4.3 Discussion of mechanisms

The efficacy of CT-guided spinal nerve root adhesiolysis in alleviating atypical CDH-associated symptoms can be attributed to multiple mechanisms closely aligned with the underlying pathophysiology.

#### 4.3.1 Core mechanism: direct relief of inflammation and compression around nerve roots

Disc herniation or osteophytes mechanically compress nerve roots, inducing sterile inflammation and subsequent dysfunction. Ozone, a potent anti-inflammatory agent, reduces inflammatory mediator release via oxidative reactions, thereby diminishing nerve root edema (7, 9, 20). Additionally, ozone’s dehydrating effect on the nucleus pulposus promotes shrinkage, directly reducing mechanical pressure on the nerve root (7). The mechanical lavage action of normal saline helps remove perineural adhesions and inflammatory products, restoring normal nerve root mobility. Local administration of anti-inflammatory solutions (e.g., betamethasone) further augments anti-inflammatory effects, rapidly alleviating nerve root irritation (21, 22). Importantly, these three components act synergistically through complementary mechanisms and temporal profiles. Ozone provides immediate biochemical modulation by oxidizing pro-inflammatory mediators and improving local oxygenation, while simultaneously offering mild mechanical decompression through its dehydrating effect on the disc material. Normal saline delivers instantaneous mechanical release by physically lavaging adhesions, fibrin deposits, and accumulated inflammatory debris, thereby restoring normal nerve root excursion. Corticosteroids sustain the anti-inflammatory effect over days to weeks by inhibiting phospholipase A2 and blocking the arachidonic acid cascade. This multimodal approach addresses both the chemical (inflammation) and mechanical (adhesion/compression) components of nerve root pathology—a distinct advantage over traditional epidural steroid injections, which target inflammation alone without addressing perineural adhesions. The combined effect is a more comprehensive and durable resolution of symptoms, as reflected in our sustained 12-month outcomes.

#### 4.3.2 Indirect regulation of autonomic nervous system and vertebral artery perfusion

Cervical nerve roots, particularly in the lower cervical spine (C5-C7), exhibit close anatomical proximity to the cervical sympathetic chain and vertebral arteries (3–5). Irritation or compression of nerve roots due to disc herniation can transmit aberrant signals to adjacent sympathetic fibers, leading to sympathetic hyperactivity or dysfunction. Sympathetic dysregulation is a significant contributor to atypical symptoms such as palpitations, gastrointestinal symptoms, tinnitus, and certain forms of dizziness (23–25). By resolving inflammation and compression around the nerve roots, CT-guided adhesiolysis effectively reduces abnormal sympathetic stimulation, stabilizing autonomic function and alleviating sympathetically mediated atypical symptoms.

#### 4.3.3 Impact on vertebral artery function

Cervical spine pathologies affecting the vertebral artery are also critical etiological factors for symptoms like dizziness and visual disturbances (3). The vertebral artery courses through the transverse foramina, and its hemodynamics can be compromised indirectly by disc herniation, osteophytes, cervical instability, or perivascular inflammation (26). Reducing perineural inflammation and releasing adhesions improves the local microcirculatory environment, potentially diminishing irritation or compression affecting the vertebral artery and enhancing perfusion. Improved vertebrobasilar blood flow is crucial for alleviating symptoms attributable to vertebral artery insufficiency.

Therefore, this therapeutic approach achieves significant clinical efficacy by targeting the complex pathophysiology of CDH with atypical symptoms through multiple integrated mechanisms: direct alleviation of nerve root inflammation and compression, indirect modulation of autonomic nervous system function, and potential improvement in vertebral artery perfusion. These combined effects underlie the overall therapeutic benefit observed with CT-guided spinal nerve root adhesiolysis.

### 4.4 Study strengths and limitations

The primary strengths of this study include its large sample size focused on a specific clinical challenge, the use of validated outcome measures, and a 12-month follow-up demonstrating sustained efficacy. However, several limitations warrant consideration. The retrospective, single-center design is susceptible to selection and information bias, and the absence of a control group precludes definitive exclusion of placebo effects or assessment of natural history in this refractory population.

Nonetheless, three observations support a genuine therapeutic effect beyond these confounders. First, all patients had symptoms refractory to ≥ 3 months of conservative treatment, making spontaneous resolution unlikely. Second, the improvement was sustained over 12 months with stable scores from 6 to 12 months—a pattern inconsistent with the typically transient nature of placebo responses. Third, subgroup analysis of patients with isolated dizziness or tinnitus (without neck pain) confirmed efficacy independent of somatic pain relief, arguing against non-specific effects. While the magnitude and persistence of improvement strongly suggest meaningful benefit, future prospective randomized controlled trials with sham or active comparators are needed to definitively quantify the treatment effect relative to natural history and placebo.

## 5 Conclusion

The findings of this study strongly suggest that CT-guided spinal nerve root adhesiolysis is an effective and safe minimally invasive option for patients with cervical disc herniation suffering from atypical symptoms. For patients who have long endured debilitating symptoms such as dizziness and tinnitus unresponsive to conservative treatments, this technique offers a targeted therapeutic solution. It not only alleviates patient suffering and improves quality of life but also provides clinicians with a valuable new tool and perspective for managing these complex cases.

## Data Availability

The original contributions presented in the study are included in the article/supplementary material, further inquiries can be directed to the corresponding authors.
